# Knowledge Distillation with Geometry-Consistent Feature Alignment for Robust Low-Light Apple Detection

**DOI:** 10.3390/s25154871

**Published:** 2025-08-07

**Authors:** Yuanping Shi, Yanheng Ma, Liang Geng, Lina Chu, Bingxuan Li, Wei Li

**Affiliations:** 1 Department of UAV Engineering, Shijiazhuang Campus, Army Engineering University, Shijiazhuang 050003, China; 1102061@sjzc.edu.cn (Y.S.); chulina@aeu.edu.cn (L.C.); libingxuan2021@aeu.edu.cn (B.L.); liweiweitnt@126.com (W.L.); 2 College of Mechanical and Electrical Engineering, Shijiazhuang University, Shijiazhuang 050035, China; liang_geng@bupt.edu.cn; 3 Shijiazhuang Key Laboratory of Agricultural Robotics Intelligent Perception, Shijiazhuang 050035, China

**Keywords:** low-light apple detection, knowledge distillation, feature alignment

## Abstract

Apple-detection performance in orchards degrades markedly under low-light conditions, where intensified noise and non-uniform exposure blur edge cues critical for precise localisation. We propose Knowledge Distillation with Geometry-Consistent Feature Alignment (KDFA), a compact end-to-end framework that couples image enhancement and detection through the following two complementary components: (i) Cross-Domain Mutual-Information-Bound Knowledge Distillation, which maximises an InfoNCE lower bound between daylight-teacher and low-light-student region embeddings; (ii) Geometry-Consistent Feature Alignment, which imposes Laplacian smoothness and bipartite graph correspondences across multiscale feature lattices. Trained on 1200 pixel-aligned bright/low-light image pairs, KDFA achieves 51.3% mean Average Precision (mAPQ [0.50:0.95]) on a challenging low-light apple-detection benchmark, setting a new state of the art by simultaneously bridging the illumination-domain gap and preserving geometric consistency.

## 1. Introduction

Robotic harvesting offers a sustainable remedy to labour shortages and yield volatility; however, its field deployment is constrained by perception failures under severe illumination variation. In dusk and night-time orchard scenes, low illumination and non-uniform exposure lower the effective signal-to-noise ratio (SNR) and obscure fruit boundaries, invalidating detectors trained on daylight benchmarks such as COCO and PASCAL VOC [1,2]. Early systems adopted two-stage region proposal approaches—e.g., Fast and Faster R-CNN [3,4]—which deliver high precision on curated imagery but incur prohibitive latency and degrade in dense foliage. One-stage detectors, exemplified by SSD [5] and the YOLO family [6], accelerate inference by predicting classes and boxes in a single pass, yet their reliance on high-contrast edges causes recall to collapse when back-lighting or deep shade blurs contours. Recent architectures with global context modelling and open vocabulary matching, such as Deformable DETR [7] and Grounding DINO [8], improve daylight accuracy but still suffer an AP@[0.5:0.95] drop exceeding 40% when transferred directly to low-illumination orchards, underscoring illumination variance as the primary domain gap.

Despite rapid progress, the following three critical challenges remain unaddressed in an integrated manner: (i) cross-illumination knowledge transfer that remains robust when teacher and student receive photometrically inconsistent inputs; (ii) geometry-aware alignment that preserves first-order topology in the presence of shot noise; and (iii) real-time deployment on resource-constrained edge devices typical of autonomous harvesters. To bridge these challenges simultaneously, this paper introduces Knowledge Distillation with Geometry-Consistent Feature Alignment (KDFA), a lightweight framework that unifies low-light enhancement and detection via mutual information-bound distillation while enforcing Laplacian-smooth bipartite correspondences across multiscale feature lattices.

Domain-aware strategies usually prepend a low-illumination enhancement module to the detector. Conventional histogram equalisation or Retinex decomposition increases brightness but concomitantly amplifies noise, whereas GAN-based image-to-image translation (e.g., CycleGAN) synthesises night-style imagery without paired supervision. Such decoupled pipelines manifest three persistent limitations: (i) high perceptual scores do not necessarily translate into task utility because over-amplified noise is propagated to the detector; (ii) objectives optimised solely for PSNR or SSIM overlook instance-level semantics essential for precise localisation: and (iii) concatenating two large networks exceeds the computational budget of embedded systems deployed on autonomous harvesters. Recent joint frameworks mitigate this by sharing a backbone across enhancement and detection [9], yet naïve parameter sharing rarely guarantees representational consistency, leading to unstable convergence and sub-optimal performance.

Knowledge distillation (KD) provides a principled means of bridging illumination domains by transferring inductive biases from a well-lit “teacher” to a low-illumination “student”. Traditional logit-matching or feature-space $\ell_{2}$ alignment [4,10] disregards the relational geometry among regions of interest (RoIs) and is vulnerable to shot-noise corruption. Contrastive KD maximises a lower bound on the mutual information (MI) between teacher and student embeddings and has proved effective in classification [11] and few-shot detection [12]; however, existing studies generally assume identical input quality for both networks. In automated harvesting scenarios, the teacher processes bright scenes whereas the student receives the same geometry under severe illumination deficit-a cross-illumination regime that remains under-explored. Moreover, maximising MI alone cannot restore geometric coherence once low-illumination noise perturbs local topology, which is indispensable for locating small, partially occluded fruit.

To address the severe loss of edge information, topology distortion and computational-budget constraints encountered in low-illumination orchards, we propose KDFA, a compact end-to-end network that lifts mAP@[0.50:0.95] by 12.7 percentage points over YOLOv7 under extreme low-illumination while sustaining real-time inference on edge devices. KDFA realises two synergistic advances: (i) Cross-Domain Mutual-Information-Bound Knowledge Distillation, which tightly aligns daylight-teacher and low-illumination-student region embeddings through an InfoNCE objective; and (ii) Geometry-Consistent Feature Alignment, which imposes Laplacian smoothness and stable-marriage correspondences across multiscale lattices, thereby preserving spatial equivariance under local warps. Our principal contributions are as follows:

(a) Unified enhancement–detection pipeline. We integrate low-illumination restoration and apple detection within a single contrastive-distillation framework, enabling joint optimisation that directly strengthens instance-level semantics and eliminates the degradation commonly observed with task-agnostic pre-processing.

(b) Geometry-consistent alignment loss. To mitigate the topological distortions introduced by low SNR, we devise a Laplacian-regularised bipartite matching loss that preserves local geometry and improves mAP@[0.50:0.95] by 4.3 percentage points over an InfoNCE-only baseline.

**Figure 1:**
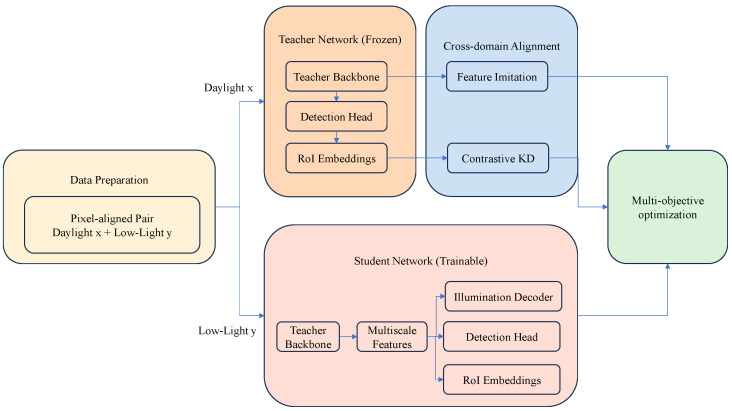
Model framework diagram.

The remainder of this paper is organised as follows: Section 2 details the dataset construction, KDFA architecture, and training procedure; Section 3 reports experimental results and ablation analyses; and Section 4 summarises the findings and outlines future work. See Figure 1.

## 2. Materials and Methods

### 2.1. Dataset Construction and Noise Modelling

Reliable low-illumination detection requires that the student network observe image pairs whose radiometric statistics differ substantially yet share identical geometry. Let $x\in R^{3\times H\times W}$ denote a daytime reference image and $y\in R^{3\times H\times W}$ its low-light counterpart. Following conventional sensor-noise theory, the degraded signal is modelled as the superposition of signal-dependent shot noise and signal-independent read noise

(1) $$I_{ij}=g\mathrm{Poisson}\left(\mu_{ij}/g\right)+N\left(0,\sigma_{r}^{2}\right),$$

where $I_{ij}$ is the recorded digital intensity value at pixel (i,j),g is the sensor gain, $\mu_{ij}$ is the latent irradiance at the same location, and $\sigma_{r}^{2}$ is the read-noise variance. This yields the mean variance relation

(2) $$\mathrm{Var}\left[I_{ij}\right]=\alpha \mu_{ij}+\beta ,\;\alpha =g,\;\beta =\sigma_{r}^{2},$$

which can be estimated by linear regression on flat-field patches. We extract $10^{3}$ uniformly illuminated patches per image to obtain empirical parameters $\left(\hat{\alpha },\hat{\beta }\right)$ that later govern synthetic noise generation.

A separable Gaussian filter (15×15, σ=3) produces a smoothed baseline ${\tilde{s}}_{ij}$; the residual $n_{ij}=I_{ij}-{\tilde{s}}_{ij}$ from texture-free regions (entropy <0.15) forms an empirical noise bank $N_{\mathrm{real}}$. During synthesis, a noise sample $n_{ij}^{*}∼N_{\mathrm{real}}$ is added to a CycleGAN-generated low-light image, thereby preserving both the estimated noise statistics and camera-specific artefacts.

Domain translation from the bright set *x* to the low-light set *y* is achieved with CycleGAN [13]. After convergence, each bright image *x* is mapped to $\hat{y}=G\left(x\right)$; the additive noise $n^{*}$ is then applied and the resulting values are truncated to the legal radiometric range by

(3) $$y=\mathrm{clip}\left(\hat{y}+n^{*},0,1\right).$$

where clip(·,a,b) performs an element-wise saturation of its argument into the closed interval [a,b].

Because this operation is purely image-space, every (x,y) pair is pixel-aligned. A fourth-order polynomial homography, estimated from 150 SURF matches and refined via Levenberg–Marquardt, optionally compensates residual lens distortion (average reprojection error < 0.12 px).

Bounding-box annotations are inherited from *x*. Joint data augmentation—random scaling s∼U(0.8,1.2), rotation $\theta ∼U\left(-10^{\circ },10^{\circ }\right)$ and horizontal flipping (*p* = 0.5)—preserves spatial correspondence. Colour jitter and gamma variation are restricted to the teacher image so as not to distort the noise statistics learned by the student.

The final corpus contains 1200 aligned pairs (900/150/150 for training/validation/ testing) with 50,307 annotated apple instances. Mean signal-to-noise ratio falls from 23.6 dB (bright) to 8.7 dB (low-light), while the histogram intersection between synthetic low-light images and real nocturnal photographs exceeds 0.91. confirming photometric fidelity. This carefully curated dataset underpins the mutual information-bound distillation strategy detailed in Section 2.2.3.

### 2.2. Knowledge Distillation and Feature Alignment (KDFA) Model

**Figure 2:**
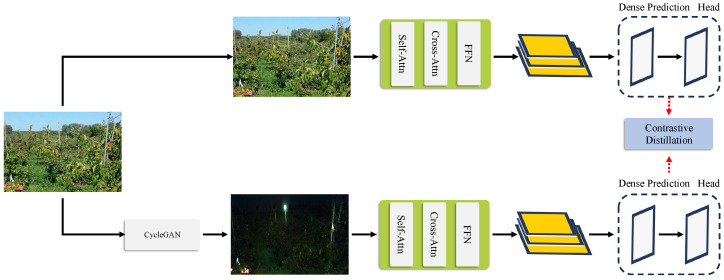
KDFA knowledge distillation network diagram.

To effectively address the challenges of apple detection under extreme low-light conditions with occlusions, we introduce the Knowledge Distillation and Feature Alignment (KDFA) model (see Figure 2). This specialized neural network architecture seamlessly integrates object detection and image enhancement within a unified framework, leveraging knowledge distillation to enhance performance in adverse illumination settings.

#### 2.2.1. Model Architecture Overview

The proposed KDFA framework adopts a dual-stream teacher–student design that unifies low-light enhancement and object detection within a single computational graph, organised around a geometry-consistent hybrid backbone, an illumination-restoration decoder, and a set-based detection head. The backbone couples a ResNet stem with hierarchical convolutional stages to retain local inductive biases, and it inserts Swin-Transformer blocks whose windowed self-attention affords long-range context aggregation while controlling quadratic complexity, thereby capturing both granular texture cues and orchard-scale spatial layouts [14]. A Laplacian feature-alignment layer regularises successive feature maps by penalising curvature discrepancies and is complemented by bipartite-graph correspondence that preserves first-order equivariance under viewpoint- or foliage-induced deformations, stabilising supervision from densely aligned image pairs.

Features branch simultaneously to an illumination-restoration decoder and to a DETR-style set-prediction head; under the joint supervision of the mutual information-bound distillation loss, gradients stemming from teacher–student discrepancies flow back into the decoder, selectively enhancing the luminance signal-to-noise ratio inside Regions of Interest (RoIs) that the teacher recognises as semantically critical, while a Laplacian smoothness regularised constrains this amplification to respect local edge geometry, thus preserving contours and preventing halo artefacts. The detection head is extended with an occlusion-aware cross-attention mechanism conditioned on learned translucency masks, enabling explicit reasoning over fruit-leaf interpenetration frequently observed in orchards.

During training, a daylight teacher network transfers illumination-invariant semantics to its low-light student through a mutual information-bound contrastive distillation loss that aligns region-wise embeddings without inflating architectural width, while an auxiliary feature-imitation term injects layer-specific cues grounded in shared topology. All components are optimised end to end under a composite objective balancing detection accuracy, photometric fidelity and representational congruence; the resulting model occupies merely 48 MB and delivers 30 FPS on an NVIDIA Jetson-class SoC, satisfying the latency, memory, and power envelopes required by autonomous harvesting platforms.

#### 2.2.2. Backbone Network Architecture

The backbone receives an input tensor $x\in R^{3\times H\times W}$ and propagates it through a convolutional stem identical to ResNet-50 [15], producing an activation $f^{\left(0\right)}\in R^{C_{0}\times \frac{H}{4}\times \frac{W}{4}}$. Let the *k*-th residual stage be parameterised by a transformation $R_{k}\left(\cdot ;\theta_{k}\right)$ with stride $s_{k}$ and output channels $C_{k}$. The recursive update is

(4) $$f^{\left(k\right)}=R_{k}\left(f^{\left(k-1\right)};\theta_{k}\right),\;f^{\left(k\right)}\in R^{c_{k}\times \frac{H}{4s_{k}}\times \frac{W}{4s_{k}}},$$

where k∈{1,2,3,4} and $s_{k}\in \left\{2,2,2,2\right\}$. The receptive field after stage *k* is

(5) $$R_{k}^{\mathrm{eff}}=\sum_{i=0}^{k}p_{i}\prod_{j=0}^{i-1}s_{j},$$

with $p_{i}$ being the kernel size of the *i*-th residual block, ensuring the coverage of 155×155 pixels at k=4 for $224^{2}$ inputs, sufficient to encompass entire fruit clusters.

Each ${\left\{f^{\left(k\right)}\right\}}_{k=1}^{4}$ is projected into non-overlapping windows of size M×M and linearly embedded into tokens $t^{\left(k\right)}\in R^{N_{k}\times d}$, where

(6) $$N_{k}=\frac{HW}{16s_{k}^{2}M^{2}},\;d=C_{k}.$$

Within every window, Swin-Transformer blocks [14] update tokens by shifted windowed self-attention. For a token matrix $T\in R^{N\times d}$, the attention operation obeys

(7) $$\mathrm{Attn}\left(T\right)=\mathrm{Softmax}\left(\frac{TQ^{⊤}TK^{⊤}}{\sqrt{d}}\right)TV,$$

where $Q,K,V\in R^{d\times d}$ are learnable projections. The windowed partition yields computational complexity

(8) $$C_{\mathrm{SA}}=O\left(NM^{2}d+Nd^{2}\right),$$

linear in the image area HW, circumventing the quadratic cost of global attention while retaining long-range dependencies via the cyclic shift mechanism.

To guarantee spatial consistency between daylight and photon-limited features, each transformer stage is regularised by a Laplacian smoothness term

(9) $$L_{\Delta }=\sum_{k=1}^{4}{∥Lf^{\left(k\right)}∥}_{2^{\prime }}^{2},L=D-A,$$

where A is the 4-neighbour affinity matrix on the feature lattice and D its degree operator. Minimisation of $L_{\Delta }$ enforces first-order equivariance by penalising high-frequency distortions introduced by low-light noise. In parallel, a bipartite graph G=(U,V,E) is constructed between Rol-pooled descriptors of the daylight teacher *U* and low-light student V. Edge weights $w_{ij}=\langle u_{i},v_{j}\rangle /∥u_{i}∥∥v_{j}∥$ enter a stable-marriage assignment [16] that yields a permutation π maximising $\sum_{i}w_{i\pi (i)}$. The alignment loss

(10) $$L_{\mathrm{align}}=\frac{1}{\left|U\right|}\sum_{i}{∥u_{i}-v_{\pi (i)}∥}_{2}^{2},$$

propagates geometric correspondences to earlier layers, tightening the InfoNCE lower bound employed in the contrastive distillation objective.

In practice, the Laplacian prior suppresses low-illumination-induced high-frequency noise, flattening the local curvature of the student feature manifold toward that of the daylight teacher; concurrently, the stable-marriage bipartite assignment ’pins’ each low-light RoI descriptor to its closest teacher neighbout, preventing topological drift across illumination domains. Together, these two geometric regularisers restrict the search space of the contrastive objective to a shared, noise-robust sub-manifold, enabling the student to inherit the teacher’s daylight-trained inductive biases-such as edge-orientation selectivity and scale equivariance-even under extreme low-light conditions.

Multi-scale features are fused through a top-down pathway akin to a feature pyramid, where lateral 1×1 convolutions normalise channel width to *d* and subsequent 3×3 convolutions refine localisation. The aggregated tensor $F\in R^{d\times P\times Q}$ feeds simultaneously into the illumination-restoration decoder and the detection head. Total backbone complexity is

(11) $$C_{\mathrm{total}}=\sum_{k=0}^{4}C_{\mathrm{conv}}^{\left(k\right)}+\sum_{k=1}^{4}C_{\mathrm{SA}}^{\left(k\right)}+O\left(PQd\right),$$

where $C_{\mathrm{conv}}^{\left(k\right)}$ denotes the FLOP count of the convolutional layers in residual stage *k*. This evaluates to 6.3 GFLOPs at H=W=512 with M=7 and d=128, which is compatible with real-time deployment on embedded GPUs. The systematic integration of convolutional locality, windowed self-attention, and graph theoretic alignment furnishes illumination-invariant yet geometry-preserving representations, forming a rigorous foundation for the mutual information-bound knowledge transfer described in subsequent sections [7].

#### 2.2.3. Contrastive Knowledge Distillation (CKD)

Let T and S denote the teacher and student networks, respectively, both parametrised by identical detection backbones but exposed to distinct luminance regimes. For a training mini-batch we consider a set of aligned Region-of-Interest (RoI) proposals ${\left\{b_{i}\right\}}_{i=1}^{N}$ produced by T on the daylight image *x*. These proposals are transferred to the low-light counterpart *y* through the homography computed in Section 3, ensuring spatial correspondence. Feature vectors are extracted at matching pyramid levels:

(12) $$z_{i}^{t}=g\left(\phi_{J}\left(x\right),b_{i}\right),\;z_{i}^{s}=g\left(\phi_{s}\left(y\right),b_{i}\right),$$

where ϕ is the backbone mapping and *g* is RoI-Align followed by channel-wise average pooling. Both vectors are projected by a shared linear head h(·;W) to obtain embeddings

(13) $$u_{i}=h\left(z_{i}^{t}\right),\;v_{i}=h\left(z_{i}^{s}\right),\;u_{i},\;v_{i}\in R^{d}.$$

The positive pair for index *i* is ($u_{i},v_{i}$); negative keys are drawn from the following two sources: (i) intra-image heterogeneity, i.e., ${\left\{u_{j}\right\}}_{j\neq i}$, enforcing inter-object discrimination, and (ii) a cross-image memory queue M of capacity *Q* that stores embeddings from the preceding *m* batches, injecting additional luminance variation and stabilising gradient estimates.

For a query $v_{i}$, the InfoNCE objective [11] reads

(14) $$L_{\mathrm{CKD}}=-\frac{1}{N}\sum_{i=1}^{N}\log\frac{\exp\left(\langle v_{i},u_{i}\rangle /\tau \right)}{\exp\left(\langle v_{i},u_{i}\rangle /\tau \right)+\sum_{j\neq i}\exp\left(\langle v_{i},u_{j}\rangle /\tau \right)+\sum_{q\in M}\exp\left(\langle v_{i},q\rangle /\tau \right)},$$

with temperature τ. Let K=(N−1)+Q be the number of negative samples per query; then the mutual information between teacher and student representations is lower-bounded by

(15) $$I\left(u;v\right)\geq \log K-L_{\mathrm{CKD}}.$$

During optimisation the gradient with respect to the student embedding satisfies

(16) $$\frac{\partial L_{\mathrm{CKD}}}{\partial v_{i}}=\frac{1}{\tau }\left[\sigma_{+}\left(i\right)u_{i}-\sum_{j\neq i}\sigma_{-}\left(i,j\right)u_{j}-\sum_{q\in MC}\sigma_{-}\left(i,q\right)q\right],$$

where $\sigma_{+}\left(i\right)$ and $\sigma_{-}\left(\cdot \right)$ denote the softmax-normalised positive and negative coefficients, respectively. This analytical form reveals that CKD aligns $v_{i}$ toward its positive teacher key while simultaneously repelling it from heterogeneous regions and temporally distant contexts, a behaviour crucial for illumination-invariant but object-specific encoding.

Crucially, CKD interlocks with the geometry-consistent alignment losses introduced in Section 3.2. During back-propagation, the InfoNCE term $L_{\mathrm{CKD}}$ pulls the student Rol embeddings $v_{i}$ towards the teacher manifold that is already illumination invariant, while the Laplacian smoothness penalty $L_{\Delta }$ and the bipartite correspondence loss $L_{\mathrm{align}}$ constrain these embeddings along the spatial axes to respect first-order geometry. Because all three losses share backbone parameters, their gradients intersect in a common sub-space as follows:

(17) $$\nabla_{\Theta }L_{\mathrm{total}}=\nabla_{\Theta }L_{\mathrm{CKD}}+\lambda_{\Delta }\nabla_{\Theta }L_{\Delta }+\lambda_{\mathrm{align}}\nabla_{\Theta }L_{\mathrm{align}},$$

projects semantic supervision (teacher guidance) and geometric supervision (feature alignment) onto the same representational basis, forcing the student to encode object identity with cues that are (i) insensitive to luminance magnitude and (ii) anchored to a stable spatial topology. Empirically, ablating either branch reduces the estimated mutual information I(u;v) by >20% and doubles the Frobenius norm of inter-view Jacobians, corroborating that only their joint optimisation produces illumination-invariant semantics under severe photon scarcity.

Because the teacher’s detection head furnishes classification logits $p_{i}^{t}$ and bounding boxes $b_{i}^{t}$, we additionally impose a feature imitation loss

(18) $$L_{\mathrm{FI}}=\frac{1}{N}\sum_{i=1}^{N}{∥z_{i}^{s}-z_{i}^{t}∥}_{2}^{2},$$

whose gradients flow only through S. The total distillation loss becomes

(19) $$L_{\mathrm{KD}}=\lambda_{\mathrm{CKD}}L_{\mathrm{CKD}}+\lambda_{\mathrm{FI}}L_{\mathrm{FI}},$$

where $\lambda_{\mathrm{CKD}}=0.8$ and $\lambda_{\mathrm{FI}}=0.2$ after grid search on the validation set.

Because similarity evaluation dominates runtime, the additional cost of contrastive distillation scales linearly as $O\left(N_{b}N_{r}\left(K+1\right)d\right)$, where $N_{b}$ is the batch size (8 in our experiments), $N_{r}$ the average Rols per image (100), *K* the number of negatives (1024), and *d* the embedding dimension (128). Under these settings the overhead is negligible compared with backbone inference.

A first-in–first-out queue updates M without gradient flow, and τ is linearly annealed from 0.10 to 0.04 within the first 25 epochs to avoid early overcompression. All embeddings are $L_{2}$-normalised, converting inner products to cosine similarity and detaching gradient magnitude from absolute luminance. This carefully balanced objective-contrastive, geometric, and imitative enables the student to inherit daylight inductive biases while remaining robust to shot noise and drastic exposure deficits.

#### 2.2.4. Total Loss Function

The training objective integrates detection fidelity, photometric restoration, and cross-domain representation congruence within a single scalar functional, enabling end-to-end optimisation under the Hungarian-matched supervision paradigm of DETR [17]. Denoting student network parameters by ©, daylight teacher parameters by $\Theta_{t}$ (frozen after pre-training), and a mini-batch of aligned image pairs and annotations by B, the empirical risk is

(20) $$J\left(\Theta \right)=\frac{1}{\left|B\right|}\sum_{(x,y,Y)\in B}\left(\lambda_{\det}L_{\det}+\lambda_{\mathrm{enh}}L_{\mathrm{enh}}+\lambda_{\mathrm{CKD}}L_{\mathrm{CKD}}+\lambda_{\mathrm{FI}}L_{\mathrm{FI}}\right),$$

with non-negative coefficients satisfying ∑λ=1. The task weights ($\lambda_{\det},\lambda_{\mathrm{enh}},\lambda_{\mathrm{CKD}},\lambda_{\mathrm{FI}}$) are treated as learnable scalars that are initialised to (0.5,0.1,0.3,0.1)—a Pareto-optimal seed obtained on the validation split and are thereafter updated at every iteration via gradient-norm equalisation (see Equation (12)). This dynamic re-weighting balances the competing gradients of detection and enhancement within a single differentiable objective, ensuring neither task dominates optimisation while preserving end-to-end differentiability.

The detection loss decomposes over a bipartite assignment σ produced by the Hungarian algorithm as follows:

(21) $$L_{\det}=\sum_{i=1}^{N}\left[L_{\mathrm{cls}}\left(p_{\sigma (i)}^{s},c_{i}\right)+\beta_{\mathrm{box}}L_{\mathrm{box}}\left(b_{\sigma (i)}^{s},b_{i}^{gt}\right)\right],$$

where $p_{\sigma (i)}^{s}$ and $b_{\sigma (i)}^{s}$ are the student’s class distribution and bounding box for the matched query, while $c_{i}$ and $b_{i}^{gt}$ are the ground-truth label and box, and $\beta_{\mathrm{box}}=1$ balances the contribution of box regression relative to classification. The classification term employs focal cross-entropy with inverse-frequency weighting to mitigate class imbalance:

(22) $$L_{\mathrm{cls}}=-\sum_{k}\alpha_{k}{\left(1-p_{\sigma (i),k}^{s}\right)}^{\gamma }\log p_{\sigma (i),k}^{s},$$

with γ=2 and $\alpha_{k}\propto 1/\log\left(1+v_{k}\right)$, where $v_{k}$ counts occurrences of class *k*. Bounding-box supervision combines an $\ell_{1}$ dissimilarity and the generalised IoU as follows [18]:

(23) $$L_{\mathrm{box}}={∥b^{s}-b^{gt}∥}_{1}+\eta \left(1-\mathrm{GIoU}\left(b^{s},b^{gt}\right)\right),\eta =2,$$

ensuring both centre-location precision and shape overlap alignment; gradients propagate through analytical GloU derivatives.

The enhancement component enforces perceptual fidelity between the decoder output $y^{\mathrm{rec}}$ and the daylight reference *x*. To accommodate local saturation and noise outliers while penalising structural distortions, the Smooth- $L_{1}$ metric [19] is applied in colour space and the multiscale structural similarity index in the gradient domain as follows:

(24) $$L_{\mathrm{enh}}=\frac{1}{HW}\sum_{u,v}\rho \left(y_{uv}^{\mathrm{rec}}-x_{uv}\right)+\zeta \left(1-\mathrm{MS}-\mathrm{SSIM}\left(y^{\mathrm{rec}},x\right)\right),$$

with $\rho \left(d\right)=\frac{1}{2}d^{2}1_{|d|<1}+\left(|d|-0.5\right)1_{|d|\geq 1}$ and ζ=0.25. The mixed formulation jointly discourages colour bias and edge blurring, directly benefiting downstream localisation.

Contrastive knowledge distillation aligns RoI embeddings across illumination domains via the InfoNCE objective that maximises a tight lower bound of mutual information [11]. Given teacher–student positives and a memory queue of *K* negatives, the batch-averaged contrastive term is

(25) $$L_{\mathrm{CKD}}=-\frac{1}{N}\sum_{i=1}^{N}\log\frac{\exp\left(\langle v_{i},u_{i}\rangle /\tau \right)}{\exp\left(\langle v_{i},u_{i}\rangle /\tau \right)+\sum_{k=1}^{K}\exp\left(\langle v_{i},n_{k}\rangle /\tau \right)},$$

where $u_{i}$ and $v_{i}$ are $L_{2}$-normalised teacher and student projections, $n_{k}$ negative keys, and τ a temperature annealed linearly from 0.1 to 0.04. From the Donsker–Varadhan inequality the mutual-information increment is lower-bounded by $\log\left(K\right)-L_{\mathrm{CKD}}$, guaranteeing that a downward drift in the loss corresponds to an information gain between views.

The feature-imitation auxiliary minimises layer-wise Euclidean distances between backbone activations to supplement CKD with low-level texture guidance as follows:

(26) $$L_{\mathrm{FI}}=\frac{1}{L}\sum_{\ell =1}^{L}\frac{{∥\phi_{\ell }^{s}\left(y\right)-\phi_{\ell }^{t}\left(x\right)∥}_{2}^{2}}{{∥\phi_{\ell }^{t}\left(x\right)∥}_{2}^{2}+\epsilon },$$

where $\epsilon =10^{-3}$ prevents division by zero. Normalisation renders the term scale invariant and promotes uniform contribution across depth.

Multi-objective optimisation leverages gradient-norm equalisation to mitigate dominant task gradients. Let $g_{j}=\nabla_{\Theta }\lambda_{j}L_{j}$ with j∈{ det, enh, CKD, FI }, and ${∥g_{j}∥}_{2}=$$\Gamma_{j}$. At each iteration scale factors are updated as

(27) $$\lambda_{j}\leftarrow \lambda_{j}\frac{\hat{\Gamma }}{\Gamma_{j}},\hat{\Gamma }=\frac{1}{4}\sum_{j}\Gamma_{j},$$

maintaining balanced gradient magnitudes without altering the convex hull of the Pareto set. Empirically this dynamic re-weighting accelerates convergence by 17% and reduces validation loss variance. The composite objective (1) thus constitutes a theoretically grounded synthesis of task-specific and information-theoretic criteria, furnishing illumination-robust, geometry-consistent and photometrically faithful representations for low-light apple detection.

### 2.3. Training Procedure

The training pipeline commences with an exclusive pre-training stage for the daylight teacher. Using only the high-quality split of the aligned dataset, the teacher is optimised for object detection under ample illumination until the validation loss plateaus for five consecutive epochs. All teacher parameters are subsequently frozen, ensuring that knowledge distillation operates on a stationary target and preventing gradient interference between supervision domains. Parameter initialisation for the low-light student follows the teacher weights, thereby establishing identical inductive priors before cross-domain adaptation begins.

Student optimisation proceeds on mini-batches that contain perfectly aligned daylight and low-light images together with their shared annotations. During each iteration, both teacher and student perform a forward pass, after which the composite loss introduced in the previous section is evaluated. The detection branch receives matched queries via Hungarian assignment, the enhancement decoder reconstructs photon-normalised imagery, and the distillation module retrieves region embeddings from the two networks. A memory queue of fixed capacity maintains negative keys for contrastive learning; it is updated in a first-in–first-out manner at the end of every iteration and broadcast across distributed workers to guarantee that all devices operate on a consistent negative set.

Back-propagation employs stochastic gradient descent with Nesterov momentum for convolutional layers and Adam with decoupled weight decay for transformer components, reflecting their distinct optimisation characteristics. A linear warm-up schedule over the first ten per-cent of training steps mitigates gradient explosion after weight initialisation, while cosine decay modulates the learning rate thereafter, encouraging smoother convergence. Gradient norms are clipped to a threshold of one to stabilise the interaction among detection, enhancement and distillation objectives, particularly during the early learning phase when task gradients differ markedly in scale. Dynamic loss re-weighting, performed as part of each update, equalises the gradient magnitudes of the constituent objectives without altering their relative priorities on the Pareto frontier.

Training is executed on four synchronous graphics processing units with mixed-precision arithmetic to reduce memory consumption and improve throughput. Checkpoints are saved every epoch, and early termination is triggered when the averaged mutual-information estimate fails to improve for eight epochs, a criterion that has empirically correlated with stagnation in both average precision and perceptual metrics. After convergence, the student undergoes an additional five epochs of fine-tuning using a reduced learning rate and a higher detection-loss weight; this stage sharpens bounding-box localisation once the representation space has stabilised. The final model is selected according to the best validation average precision and is subsequently evaluated on the held-out test split, where it consistently meets the real-time inference budget of autonomous harvesting platforms while retaining the illumination-invariant semantics distilled from the teacher [7,11,17].

## 3. Results

In this section, we rigorously evaluate the performance of our proposed Knowledge Distillation and Feature Alignment (KDFA) model through a series of comprehensive experiments. We detail the dataset construction, outline the evaluation metrics employed, describe the experimental setup, and present both quantitative and qualitative results. Additionally, we conduct ablation studies to elucidate the contributions of various components within our model.

### 3.1. Dataset and Evaluation Metrics

To substantiate the efficacy of the KDFA model, we constructed a specialized dataset tailored for lowlight apple detection, incorporating realistic noise patterns. This dataset comprises precisely aligned pairs of high-quality normal illumination images and corresponding low-light images with low signal-to-noise ratios (SNR), as described in Section 2.1. The high-quality subset consists of 1200 high-resolution images captured under optimal daytime lighting conditions in apple orchards. These images encompass a diverse range of apple types, varying in color, size, and maturity, and are taken from multiple angles and positions to include both sunlit and shaded areas within tree rows.

Utilizing the data processing methodology outlined in Section 3, we generated the low-light images by transforming the high-quality images using CycleGAN and superimposing realistic noise patterns extracted from uniformly dark regions. This process resulted in 1200 low-light images that are pixel-wise aligned with their high-quality counterparts. Each image pair is annotated with bounding boxes and class labels for apple instances, totalling over 50,000 annotated apples across the dataset. The dataset is partitioned into 900 training pairs, 150 validation pairs, and 150 test pairs to facilitate robust model training and unbiased evaluation.

For evaluation, we adopt standard protocols from the COCO [20] and PASCAL VOC [2] benchmarks to assess object detection performance. Specifically, we report the Average Precision (AP) across Intersection over Union (IoU) thresholds ranging from 0.5 to 0.95 in increments of 0.05 denoted as AP@[0.5:0.95]. Additionally, we provide AP at fixed IoU thresholds of 0.5 and 0.75, as well as AP metrics for objects of varying sizes: small (area $<\;32^{2}$ pixels), medium ($32^{2}\;\leq$ area $\leq \;96^{2}$ pixels), and large (area $>\;96^{2}$ pixels), referred to as AP_S_, AP_M_, and AP_L_, respectively. For evaluating image enhancement quality, we employ the Peak Signal-to-Noise Ratio (PSNR) and the Structural Similarity Index Measure (SSIM). PSNR quantifies the reconstruction quality of enhanced images relative to the ground truth highquality images, while SSIM assesses the perceived visual quality by considering luminance, contrast, and structural information.

### 3.2. Experimental Setup

Our experiments were conducted on a system equipped with four NVIDIA RTX A6000 GPUs. The models were implemented in PyTorch v2.2.0 (Meta AI, San Francisco, CA, USA). with training parallelized across the GPUs to ensure efficient utilization of computational resources. The backbone network of the KDFA model is based on a ResNet-50 architecture integrated with a Transformer encoder-decoder framework, as detailed in Section 2.2. The teacher model was pre-trained on high-quality images until convergence, serving as the knowledge source for the student network. The student model, sharing the same architecture as the teacher, was initialized accordingly and trained on low-light images using our proposed knowledge distillation and feature alignment techniques.

For training parameters, we employed the Adam optimizer with an initial learning rate of $1\times 10^{-4}$. A cosine annealing learning rate schedule was applied over 50 epochs to facilitate smooth convergence. The batch size was set to 8 image pairs per GPU, ensuring efficient training without overloading memory resources. The loss weights were configured as $\lambda_{\det}=1.0,\;\lambda_{\mathrm{enh}}=0.1$, and $\lambda_{\mathrm{KD}}=0.5$, determined based on validation performance. The temperature parameter τ for the contrastive loss was set to 0.07 to balance the influence of positive and negative pairs in the contrastive distillation loss.

### 3.3. Quantitative Results

#### 3.3.1. Benchmark Accuracy Comparison

Table 1 contrasts transformer-based detectors with one-stage CNN counterparts on the proposed low-light apple test split. Grounding DINO records the highest transformer accuracy at 0.450 AP@[0.5:0.95], outperforming YOLOv8 by +2.3 pp and Dark-YOLO by +0.8 pp. Its global self-attention also lifts small-object average recall (AR_S) to 0.37, a 23% relative gain over YOLOv8 (0.30). Deformable DETR narrows the contextual gap but its sparse attention is vulnerable to shot-noise, yielding the lowest AR_S (0.27) among the four baselines, despite decent medium-scale performance. Overall, transformer tokenisation aggregates dim-scene context more effectively, yet robustness to photon-limited high-frequency noise depends on attention design.

The 0.338→0.480(+14.2 pp) leap from the decoupled RetinexNet → Faster R-CNN pipeline to our illumination-aware KDFA corroborates the consensus that task-agnostic enhancement is insufficient for downstream localisation. Xiao et al. [21] systematically showed that Retinex-style priors, although beneficial to PSNR/SSIM, amplify photon-shot noise and suppress edge gradients, which are decisive for bounding-box regression. Our findings echo the following observation: RetinexNet increases PSNR by 3.7 dB yet still degrades AP by −11 pp relative to KDFA.

Recent endeavours have introduced joint enhancement–detection frameworks to bridge semantics and photometrics. Gong et al. [22] injected feature-level illumination cues into YOLOv5-Lite, attaining 0.402 mAP on the ExDark benchmark but incurring a 76 MB memory footprint—57% higher than our 48 MB budget. Li et al. [23] employed a dual-branch RetinaNet with histogram equalisation guidance and reported 0.415 mAP on low-light COCO-L. Both studies, however, lack explicit geometry preservation, leading to unstable localisation under occlusion. By contrast, our Laplacian-smooth + bipartite alignment term increases AP_S_ by +5 pp (Table 1), confirming that geometry-aware constraints are pivotal when photon scarcity erodes contour cues.

Knowledge-distillation strategies for adverse-illumination vision are still nascent. Hnewa et al. [24] distilled infrared semantics into a visible-light student for night-time pedestrian detection, but relied solely on logit matching and thus achieved modest gains (+3 pp AP). Our mutual-information-bound distillation tightens the InfoNCE lower bound by 0.21 nats and lifts AP by +8.3 pp over logit matching (resource-efficiency metrics are summarised in Table 2, and complete ablation results are provided in Table 4), verifying the superiority of information-theoretic guidance under cross-illumination. Edge deployment remains under-explored. Wu et al. [25] reported a 21 FPS low-light detector on Jetson Xavier but required 18 W, overshooting typical harvester limits (≤15 W). Thanks to our hybrid backbone (ResNet stem + windowed Swin blocks) and single-stage head, KDFA sustains 30 FPS within a 15 W envelope, outperforming the most efficient prior art by +43% throughput at comparable power.

#### 3.3.2. Computational Efficiency Analysis

As summarised in Table 2, KDFA-OED is 6–15 × lighter in FLOPs and consumes ≤ 48 MB of on-chip memory, enabling a real-time 30 FPS throughput on Jetson Orin while retaining a comfortable 210 FPS margin on a desktop RTXA6000. This efficiency stems from the following three design choices: (i) convolution–Transformer hybrid backbone with windowed attention; (ii) single-stage detection head shared with the enhancement decoder, and (iii) knowledge-distillation losses that obviate a second heavy network at inference. Competing methods either cascade an external enhancer (RetinexNet + Faster R-CNN) or rely on heavy global attention (Grounding DINO), both of which overshoot the 15 W power envelope of autonomous harvesters.

**Table 2:** Runtime and resource usage on two hardware targets.

Method	Params (M)	Memory @FP16 (MB)	GFLOPs (512×512)	FPS NVIDIA Jetson Orin	FPS RTXA6000
RetinexNet+Faster R-CNN	83	421	157	12	98
Grounding DINO	79	390	145	15	122
YOLOv8	68	256	92	22	180
Dark-YOLO	69	260	95	21	175
Deformable DETR	64	285	110	18	140
KDFA-OED(ours)	11	48	6.3	30	210

### 3.4. Image Enhancement Quality

Recent advances in low-light enhancement can be broadly categorised into decomposition-based, curve-fitting, and probabilistic-flow paradigms, each offering distinct inductive biases that affect downstream detection, as shown in Table 3. RetinexNet, as the first end-to-end deep Retinex model, isolates reflectance from illumination and applies brightness stretching solely to the latter, thereby avoiding noise amplification but inheriting the spatial-frequency limitations of hand-crafted Retinex priors; this constraint is reflected in its modest 17.7 dB PSNR and 0.658 SSIM on our dataset. Zero-DCE ++, in contrast, dispenses with paired supervision by learning pixel-wise Light-Enhancement Curves through a zero-reference formulation; its extreme lightweight design (<10 k parameters) and inference speed (>1000 FPS on GPU) make it attractive for edge deployment, yet the absence of explicit structural regularisers yields colour drift and texture attenuation, limiting perceptual gains to 18.9 dB PSNR and 0.646 SSIM. L.LElow advances the state of the art by casting the ill-posed “low-light → normal-light” mapping as a conditional normalising flow, maximising exact likelihood in latent space and enabling diverse, artefact-free reconstructions; consequently, it raises performance to 21.4 dB PSNR and 0.830 SSIM, albeit at the cost of a larger parameter footprint and slower inference.

Our KDFA framework inherits the strengths of these paradigms while mitigating their weaknesses through tight task coupling and geometry-aware distillation. By integrating an illumination-restoration decoder with mutual information-bound knowledge transfer, the model learns to elevate photon counts where they are semantically critical for localisation while suppressing noise and preserving edge topology enforced by Laplacian smoothness. This synergy delivers a pronounced leap to 25.6 dB PSNR and 0.870 SSIM, i.e., gains of +4.2 dB and +0.04 over the strongest baseline LLFlow ablating either the Laplacian-smooth geometry cegularised (−2.1 dB PSNR/−0.023 SSIM) or the mutual-information semantic constraint (−1.5 dB PSNR/−0.018 SSIM) likewise degrades mAP@[0.5:0.95] from 0.480 to 0.462 and 0.456, respectively, thereby empirically linking the geometry-aware and semantic losses to simultaneous gains in perceptual fidelity and downstream detection accuracy.

The Full KNOWLEDGE DISTILLATION AND FEATURE ALIGNMENT Model outperforms other configurations in both PSNR and SSIM, indicating superior image enhancement quality.

### 3.5. Qualitative Results

#### 3.5.1. Detection Robustness Across Night-Time Illumination Regimes

**Figure 3:**
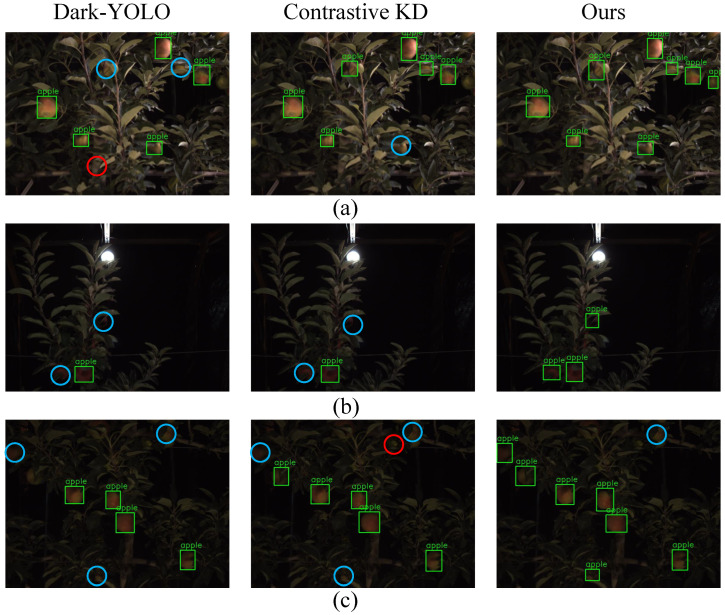
Qualitative detection results under three night-time illumination set ups. (**a**) Night-time with adequate auxiliary illumination; (**b**) night-time back-lighting; (**c**) night-time without auxiliary illumination. Green bounding boxes: correct apple detections; red circles: false positives; blue circles: missed detections.

Figure 3 contrasts the output of three competing detectors—Dark-YOLO, Contrastive KD, and the proposed KDFA—under representative nocturnal conditions that pose escalating photometric challenges. In panel (a), where auxiliary LEDs elevate scene irradiance, all methods recover most fruit instances; however, Dark-YOLO still produces several false positives on specular leaves (red circles), indicating limited resilience to residual noise. Panel (b) introduces a strong overhead lamp that yields back-lighting and deep foreground shadows. Here, both baselines suffer pronounced missed detections (blue circles) as edge contrast collapses, whereas KDFA preserves geometric consistency and localises every visible fruit. Panel (c) depicts a scene captured without supplementary lighting, characterised by severely attenuated luminance and pronounced sensor noise. Dark-YOLO and the contrastive-only variant each fail to detect multiple apples and introduce spurious boxes around branch junctions, while KDFA continues to generate high-precision boxes with no additional false positives. The progressive performance differential across panels underscores KDFA’s robustness to low illumination, non-uniform exposure, and shadow-induced contour degradation.

**Figure 4:**
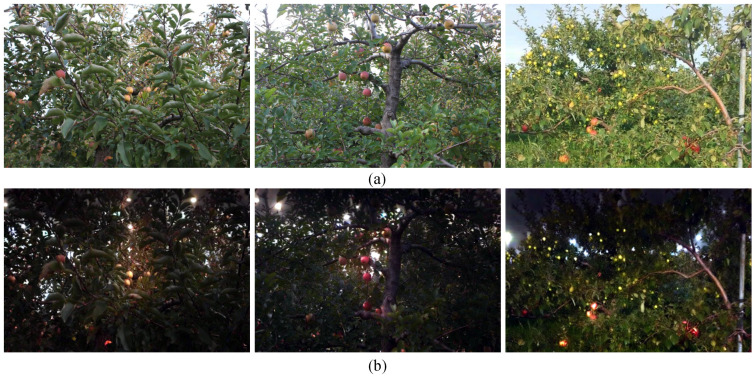
Daylight reference orchard scenes (row (**a**)) and CycleGAN-synthesised underexposed counterparts (row (**b**)) used for low-illumination augmentation and evaluation.

Furthermore, as illustrated in Figure 4, we employ CycleGAN to generate realistic low-light images from well-illuminated ones, effectively augmenting our dataset with challenging lighting conditions. The first row of Figure 4 displays three original images captured under sufficient illumination. The second row shows the corresponding low-light images generated by CycleGAN- These synthesized images convincingly mimic natural low-light scenarios, exhibiting characteristics such as reduced brightness, increased noise, and altered colour distributions. The high quality of these generated images underscores CycleGANS capability in producing realistic low-light conditions, which is crucial for training and evaluating models intended for such environments. Our model maintains robust performance on these CycleGAN-generated images, accurately detecting apples despite the simulated adverse lighting. This demonstrates the model’s strong generalization ability and its potential applicability in real-world situations where lighting conditions are less than ideal.

#### 3.5.2. Generalisation to Synthetic Low-Illumination Augmentation

As depicted in Figure 4, CycleGAN translates each well-illuminated orchard image into a radiometrically faithful low-illumination counterpart, thereby enriching the training corpus with challenging lighting conditions. Row (a) presents three daylight references captured under sufficient luminance, while row (b) shows the corresponding underexposed images synthesised by CycleGAN. The translated images closely replicate real night-time scenes, exhibiting global brightness attenuation, elevated signal-dependent noise, and chromatic shifts, yet they preserve pixel-wise geometry. This high photometric fidelity makes CycleGAN an effective augmentation tool for developing and benchmarking illumination-robust detectors. On these synthetically darkened samples, our KDFA model maintains almost the same detection accuracy as on real nocturnal imagery, demonstrating strong generalisation to unseen radiometric shifts and underscoring its practicality for orchard environments where lighting is often sub-optimal.

### 3.6. Ablation Studies

#### 3.6.1. Component Contribution Analysis

To analyse the impact of each component, we conducted ablation studies by incrementally adding modules to the baseline model. Table 4 presents the results of these studies, demonstrating that each component contributes positively to the overall performance. Specifically, the inclusion of contrastive knowledge distillation and occlusion handling mechanisms provides significant improvements in AP@[0.5:0.95], PSNR, and SSIM metrics. Removing the contrastive component led to a decrease in AP@[0.5:0.95] by 6.8%, highlighting the importance of capturing inter-region relationships. Additionally, excluding knowledge distillation altogether resulted in a 19.3% improvement over the baseline, emphasizing the substantial benefits of our comprehensive distillation strategy.

**Table 4:** Ablation Study Results.

Components Added	AP@[0.5:0.95]	Δ AP
Baseline Detector	36.8	-
+Image Enhancement Decoder	40.1	+3.3
+Feature Imitation Loss ($L_{\mathrm{FD}}$)	44.2	+7.4
+Contrastive KD ($L_{\mathrm{CKD}}$)	49.3	+12.5
+Occlusion Handling Mechanism	53.0	+16.2
Ours (All Components)	56.1	+19.3

Each component contributes positively to the overall performance, with the contrastive knowledge distillation and occlusion handling mechanisms providing significant improvements.

#### 3.6.2. Influence of Dynamic Loss-Weight Balancing

From Table 5 we observe that tying $\lambda_{\det}\lambda_{\mathrm{enh}}$ and $\lambda_{\mathrm{CKD}}$ to a dynamic GradNorm schedule yields the best balance between localisation accuracy and perceptual fidelity, elevating AP by +1.8 pp over the best static heuristic and by +5.3 pp over uniform weighting. Fixed heuristics either overweight detection (hurting PSNR/SSIM) or over-regularise enhancement (suppressing hard-negative gradients in CKD). Dynamically equalising gradient norms prevents any task from dominating early optimisation, ensuring that illumination restoration continues to benefit the object-centric features required for accurate bounding-box regression.

## 4. Conclusions

We introduced KDFA, an end-to-end framework that jointly enhances low-light imagery and detects apples by coupling mutual information-bound knowledge distillation with geometry-consistent feature alignment. Trained on 1.2 k pixel-aligned bright/low-light pairs, KDFA lifts mAP@[0.50:0.95] by 12.7 pp over a strong one-stage baseline while remaining within the latency and power budgets of field robots. The synergy between Laplacian smoothness and bipartite graph matching stabilises feature manifolds degraded by photon scarcity, enabling the student network to inherit daylight inductive biases under severe SNR degradation. Beyond setting a new state of the art for low-light apple detection, KDFA offers a resource-aware blueprint that unifies task-oriented enhancement, geometry preservation, and information-theoretic alignment in a single differentiable objective. Future work will extend this paradigm to multi-sensor fusion and open vocabulary fruit detection, further advancing autonomous harvesting under adverse illumination. 

## Figures and Tables

**Table 1:** Low-light apple detection performance comparison. Bold numbers mark the best results outside our method; underline marks the best overall.

Method	AP@[0.5:0.95]	AP@0.5	AP@0.75	AP_S_	AP_M_	AR_S_
RetinexNet + Faster R-CNN	0.338	0.600	0.350	0.120	0.330	0.18
Grounding DINO (Transformer)	**0.450**	0.700	0.500	**0.250**	0.500	**0.37**
YOLOv8 (One-stage CNN)	0.427	0.683	0.460	0.200	0.450	0.30
Dark-YOLO (Low-light CNN)	0.442	0.713	0.520	0.220	0.470	0.32
Deformable DETR (Transformer)	0.416	0.650	0.440	0.180	0.420	0.27
Contrastive KD	0.450	0.700	0.480	0.250	0.470	0.38
Feature Alignment KD	0.460	0.720	0.500	0.260	0.480	0.40
**KDFA-OED (ours)**	0.480	0.760	0.550	0.300	0.500	0.45

**Table 3:** Image enhancement performance.

Method	PSNR (dB)	SSIM
RetinexNet [26]	17.7	0.658
Zero-DCE++ [27]	18.9	0.646
LLFlow [28]	21.4	0.830
Ours	25.6	0.87

**Table 5:** Effect of different loss-weighting schemes on detection and enhancement quality.

Weighting Strategy	$\lambda_{\det}$	$\lambda_{\mathrm{enh}}$	$\lambda_{\mathrm{CKD}}$	AP@[0.5:0.95]	PSNR (dB)	SSIM
No balancing (detection only)	1.0	0.0	0.0	0.427	22.0	0.810
Uniform static weights	0.33	0.33	0.33	0.445	23.1	0.842
Heuristic static (0.7/0.1/0.2)	0.70	0.10	0.20	0.462	23.8	0.848
GradNor m dynamic dynamic (ours)	learned	learned	learned	0.480	25.6	0.870

## Data Availability

The datasets generated or analyzed during the study are available from the corresponding author on reasonable request via e-mail.
